# Comparison of 16S rRNA Gene Based Microbial Profiling Using Five Next-Generation Sequencers and Various Primers

**DOI:** 10.3389/fmicb.2021.715500

**Published:** 2021-10-14

**Authors:** Changwoo Park, Seung Bum Kim, Sang Ho Choi, Seil Kim

**Affiliations:** ^1^Department of Agricultural Biotechnology, Seoul National University, Seoul, South Korea; ^2^Group for Biometrology, Korea Research Institute of Standards and Science, Daejeon, South Korea; ^3^Center for Convergent Research of Emerging Virus Infection, Korea Research Institute of Chemical Technology, Daejeon, South Korea; ^4^Department of Biological Sciences, College of Bioscience and Biotechnology, Chungnam National University, Daejeon, South Korea; ^5^Center for Food and Bioconvergence, Seoul National University, Seoul, South Korea; ^6^Department of Bio-Analysis Science, University of Science and Technology, Daejeon, South Korea

**Keywords:** 16S rRNA, next-generation sequencing (NGS), Droplet Digital PCR (ddPCR), Bias Index (BI), mock community, reference material (RM)

## Abstract

Microbial community analysis based on the 16S rRNA-gene is used to investigate both beneficial and harmful microorganisms in various fields and environments. Recently, the next-generation sequencing (NGS) technology has enabled rapid and accurate microbial community analysis. Despite these advantages of NGS based metagenomics study, sample transport, storage conditions, amplification, library preparation kits, sequencing, and bioinformatics procedures can bias microbial community analysis results. In this study, eight mock communities were pooled from genomic DNA of *Lactobacillus acidophilus* KCTC 3164^T^, *Limosilactobacillus fermentum* KCTC 3112^T^, *Lactobacillus gasseri* KCTC 3163^T^, *Lacticaseibacillus paracasei* subsp. *paracasei* KCTC 3510^T^, *Limosilactobacillus reuteri* KCTC 3594^T^, *Lactococcus lactis* subsp. *lactis* KCTC 3769^T^, *Bifidobacterium animalis* subsp. *lactis* KCTC 5854^T^, and *Bifidobacterium breve* KCTC 3220^T^. The genomic DNAs were quantified by droplet digital PCR (ddPCR) and were mixed as mock communities. The mock communities were amplified with various 16S rRNA gene universal primer pairs and sequenced by MiSeq, IonTorrent, MGIseq-2000, Sequel II, and MinION NGS platforms. In a comparison of primer-dependent bias, the microbial profiles of V1-V2 and V3 regions were similar to the original ratio of the mock communities, while the microbial profiles of the V1-V3 region were relatively biased. In a comparison of platform-dependent bias, the sequence read from short-read platforms (MiSeq, IonTorrent, and MGIseq-2000) showed lower bias than that of long-read platforms (Sequel II and MinION). Meanwhile, the sequences read from Sequel II and MinION platforms were relatively biased in some mock communities. In the data of all NGS platforms and regions, *L. acidophilus* was greatly underrepresented while *Lactococcus lactis* subsp. *lactis* was generally overrepresented. In all samples of this study, the bias index (BI) was calculated and PCA was performed for comparison. The samples with biased relative abundance showed high BI values and were separated in the PCA results. In particular, analysis of regions rich in AT and GC poses problems for genome assembly, which can lead to sequencing bias. According to this comparative analysis, the development of reference material (RM) material has been proposed to calibrate the bias in microbiome analysis.

## Introduction

A microbiome is a microbial community of occupied habitat with physical and chemical feature (Berg et al., 2020). There are more than 10^14^ ∼ 10^15^ microorganisms present in the human body, a number 2 ∼ 3 times greater than that of human cells (Sender et al., 2016). The number of microbial genes in the human body is known to be more than 100 times the number of human genes (Gill et al., 2006; Gilbert et al., 2018). Among the microorganisms in the body, gut microbes have tremendous potential to affect our body, in terms of both health and disease. Within the gastrointestinal tract, they contribute to protection against pathogens, maintain homeostasis and metabolic functions, and improve the immune system and affect most of our body functions (Round and Mazmanian, 2009; Varian et al., 2016).

Intestinal microorganisms not only break down complex carbohydrates and fiber but also produce short-chain fatty acids (SCFAs) such as acetate, propionate, and butyrate in the gut (Den Besten et al., 2013). SCFAs lower the pH of the intestine, which interrupts the activity of pathogens and improves nutrient absorption (Macfarlane and Macfarlane, 2012; Arun et al., 2019). In the past few decades, it has become apparent that SCFAs from the gut microbiome might play an important role in the prevention and cure of metabolic syndrome bowel disorders as well as in the treatment of ulcerative colitis and Crohn’s disease (Den Besten et al., 2013).

Lactic acid bacteria (LAB) share a long and intricate history with human life (Douillard and de Vos, 2014). Many of the LABs are probiotic strain (Klein et al., 1998). The probiotics are defined as “live microorganisms which when administered in adequate amounts confer a health benefit on the host” (Fijan, 2014). The probiotics interact with the intestinal microbiome. For example, several studies have shown that the use of probiotics is associated with a reduced risk of antibiotic-related diarrhea. The use of antibiotics can affect the balance of beneficial and harmful bacteria in the gut. Researchers found that the administration of probiotics reduced antibiotic-related diarrhea (Susanne Hempel et al., 2012).

Identifying the relationship between gene distribution and the function of microbes in the human body is an important research topic (Human Microbiome Jumpstart Reference Strains Consortium et al., 2010). The importance of microbiomes thus has been increasingly recognized, and a great deal of research to analyze them is ongoing (Turnbaugh et al., 2009). Recently, targeted amplicon-based metagenome sequencing using the 16S rRNA gene has be used to research complex microbial communities such as the human intestinal microbiome (Fettweis et al., 2012; Plummer and Twin, 2015). However, despite the advantage of NGS based metagenomics study, various biases can occur throughout the workflow. In particular transport and storage conditions of environmental samples, the DNA extraction methods, and PCR can introduce bias (Boers et al., 2019). During sequencing, strong sequencing bias can be introduced by imbalance of GC contents, storage conditions, and protocols (Laursen et al., 2017). In addition, the sequencing results can be biased by library preparation for NGS, the process of various sequencing platforms, and by different bioinformatics procedures (Poretsky et al., 2014; Plummer and Twin, 2015).

In this study, eight probiotics strains were selected from the list of the government announcement for food and drug by Ministry of Food and Drug Safety in Korea and pooled to eight mock communities. The nineteen probiotics strains of the government announcements were listed on Supplementary Table 1. A comparison of amplicon bias from MiSeq (Illumina, United States), Sequel II (Pacific Biosciences, United States), IonTorrent (Thermo Fisher Scientific, United States), MGIseq-2000 (BGI, China), and MinION (Oxford Nanopore, United Kingdom) sequencing platforms was done using the MOTHUR pipeline based on following step; remove the unnecessary, alignment, classification, calculate the sequencing error (López-García et al., 2018). The sequencing results of the V1-V2 region (27F-337R), V3 region (337F-518R), V4 (518F-800R) region, and V1-V3 region (27F-518R) primer sites also were compared by using the MOTHUR pipeline. On the basis of the results, the development of a reference material (RM) is proposed to calibrate the bias in microbiome analysis (Hardwick et al., 2018; McLaren et al., 2019).

## Materials and Methods

### DNA Extraction

The strains of *Lactobacillus acidophilus* KCTC 3164^T^, *Limosilactobacillus fermentum* KCTC 3112^T^, *Lactobacillus gasseri* KCTC 3163^T^, *Lacticaseibacillus paracasei* subsp. *paracasei* KCTC 3510^T^, *Limosilactobacillus reuteri* KCTC 3594^T^, *Bifidobacterium*. *Animalis* subsp. *lactis* KCTC 5854^T^, *Bifidobacterium breve* KCTC 3220^T^, and *Lactococcus lactis* subsp. *lactis* KCTC 3769^T^ were obtained from Korean Collection for Type Cultures (KCTC) (Table 1). The detailed information of the strains is summarized in Table 1. The strains of *Lactobacillus* were cultured using MRS medium (Difco, United States) at 37°C in an aerobic condition. The *L*. *lactis* was cultured using MRS medium at 30°C in an aerobic condition. The strains of *Bifidobacterium* were cultured using MRS medium at 37°C in anaerobic condition based on the following criteria: H_2_, 4%; CO_2_, 10%; and N_2_, balance. Eight strains were cultured in 2 mL MRS broth at 48 h, according to each culture collection’s instructions. The cells were harvested with centrifugation at 13,000 rpm for 5 min from 2 mL of broth culture. The genomic DNAs were extracted using a GenElute Bacterial Genomic DNA extraction kit (Sigma-Aldrich, United States), according to the manufacturer’s instructions.

**TABLE 1:** List of probiotic strains obtained from Korean Collection for Type Cultures (KCTC).

Taxonomy	KCTC number	Number of contigs	Accession number	GC (%)	16S rRNA copy number	Genome size (Mb)
*Lactobacillus acidophilus*	KCTC 3164^T^	1	GCA_003047065.1	34.7	4	2.00997
*Limosilactobacillus fermentum*	KCTC 3112^T^	1	GCA_000010145.1	51.5	5	2.09868
*Lactobacillus gasseri*	KCTC 3163^T^	1	GCA_000014425.1	35.3	6	1.89436
*Lacticaseibacillus paracasei* subsp. *paracasei*	KCTC 3510^T^	3	GCA_000829035.1	46.6	5	3.01780
*Limosilactobacillus reuteri*	KCTC 3594^T^	1	GCA_000010005.1	38.9	6	2.03941
*Bifidobacterium animalis* subsp. *lactis*	KCTC 5854^T^	1	GCA_000022965.1	60.5	4	1.93269
*Bifidobacterium breve*	KCTC 3220^T^	1	GCA_900637145.1	58.9	3	2.26941
*Lactococcus lactis* subsp. *lactis*	KCTC 3769^T^	3	ATCC 19435*	35.4	6	2.62782

*^T^Type strain.*

*ATCC genome portal.

### Genomic DNA Quantification

The concentration of purified genomic DNA was initially determined using a QuantiFluor dsDNA System and Quantus^TM^ Fluorometer (Promega, United States) according to the manufacturers’ instructions. After measuring the concentration of genomic DNA, the copy number of genomic DNA was determined using a QX200 Droplet Digital PCR (ddPCR) system (Bio-Rad, United States) according to the manufacturers’ instructions. Primers used for ddPCR is shown in Table 2. Next, 1 μl of template, 10 μl of 2× QX200 ddPCR EvaGreen Supermix (Bio-Rad, United States), 0.5 μl of 337F forward primer, 0.2 μl of 518R reverse primer, and 8.3 μl of distilled water were mixed (Moreno et al., 2002). A total of 20 μl of mixture and 70 μl of Droplet Generation Oil for Probes (Bio-Rad, United States) were used to make 45 μl of droplet template using a QX200 Droplet Generator (Bio-Rad, United States). Amplification was then performed via initial denaturation at 95°C for 5 min, followed by 40 cycles of denaturation at 95°C for 30 s, primer annealing and extension at 60°C for 1 min, and then one cycle of signal stabilization at 4°C for 5 min and 90°C for 5 min. All thermal cycling experiments were performed on a Veriti Thermal Cycler (Thermo Fisher, United States) using a ramp rate of 2°C/s (Miotke et al., 2015). The fluorescence was initially analyzed using QuantaSoft software (Bio-Rad, United States). The copy numbers of the genomic DNAs were summarized (Supplementary Table 2).

**TABLE 2:** Primer sequence used for PCR, ddPCR, and NGS in this study.

Platforms	Primer name	Oligonucleotide sequence (5′–3′)	Purpose
QX 200	337F	GACTCCTACGGGAGGCWGCAG	ddPCR quantification
	518R	CGTATTACCGCGGCTGCTGG	
MiSeq	MiSeq_27F	TCGTCGGCAGCGTC-AGATGTGTATAAGAGACAG-AGAGTTTGATCMTGGCTCAG	Genomic DNA amplicon
	MiSeq_bif27F	TCGTCGGCAGCGTC-AGATGTGTATAAGAGACAG-GGGTTCGATTCTGGCTCAG	
	MiSeq_337R	GTCTCGTGGGCTCGG-AGATGTGTATAAGAGACAG-CTGCWGCCTCCCGTAGGAGTC	
	MiSeq_337F	TCGTCGGCAGCGTC-AGATGTGTATAAGAGACAG-GACTCCTACGGGAGGCWGCAG	
	MiSeq_518R	GTCTCGTGGGCTCGG-AGATGTGTATAAGAGACAG-CGTATTACCGCGGCTGCTGG	
	MiSeq_518F	TCGTCGGCAGCGTC-AGATGTGTATAAGAGACAG-CCAGCAGCCGCGGTAATACG	
	MiSeq_800R	GTCTCGTGGGCTCGG-AGATGTGTATAAGAGACAG-TACCAGGGTATCTAATCC	
	I5 forward primer	AATGATACGGCGACCACCGAGATCTACAC-XXXXXXXX-TCGTCGGCAGCGTC	Nextera barcoding
	I7 reverse primer	CAAGCAGAAGACGGCATACGAGAT-XXXXXXXX-GTCTCGTGGGCTCGG	
IonTorrent	27F	AGAGTTTGATCMTGGCTCAG	Genomic DNA amplicon
	bif27F	GGGTTCGATTCTGGCTCAG	
	337R	CTGCWGCCTCCCGTAGGAGTC	
	337F	GACTCCTACGGGAGGCWGCAG	
	518R	CGTATTACCGCGGCTGCTGG	
	518F	CCAGCAGCCGCGGTAATACG	
	800R	TACCAGGGTATCTAATCC	
MGIseq-2000	337F	GACTCCTACGGGAGGCWGCAG	
	518R	CGTATTACCGCGGCTGCTGG	
Sequel II MinION	27F	AGAGTTTGATCMTGGCTCAG	
	bif27F	GGGTTCGATTCTGGCTCAG	
	1492R	TACGGYTACCTTGTTACGACTT	

### Construction of Mock Community

**FIGURE 1:**
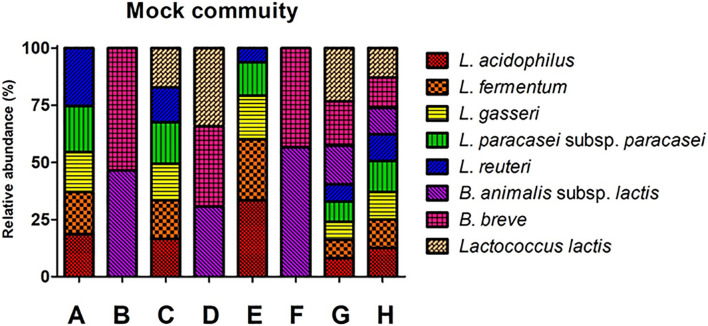
Structures of eight mock communities based on copy number. The percent number in the graph indicates the ratio of each community. A and E: *Lactobacillus* group, B and F: *Bifidobacterium* group, C: *Lactobacillus* and *Lactococcus* group, B: *Bifidobacterium* and *Lactococcus* group, G and H: *Lactobacillus, Bifidobacterium* and *Lactococcus* group. The *X*-axis shows the mock community. The *Y*-axis indicates relative abundance.

Mock communities were made using the genomic DNA from eight probiotic strains. The ratio of the genomic DNA in the mock communities was determined based on the ddPCR (Supplementary Table 2). The mock community consists of eight groups: A, B, C, D, E, F, G, and H (Figure 1). Mock A, B, C, D, and H were designated as mock communities with an even ratio, while mock E, F, and G were designed as mock communities with an odd ratio (Supplementary Table 2). The copy number of each genomic DNA in the mock community was determined based on the dilution rate of the diluted sample for ddPCR and the input volume of each strain.

### 16S rRNA Gene Amplification of the Mock Community

**FIGURE 2:**
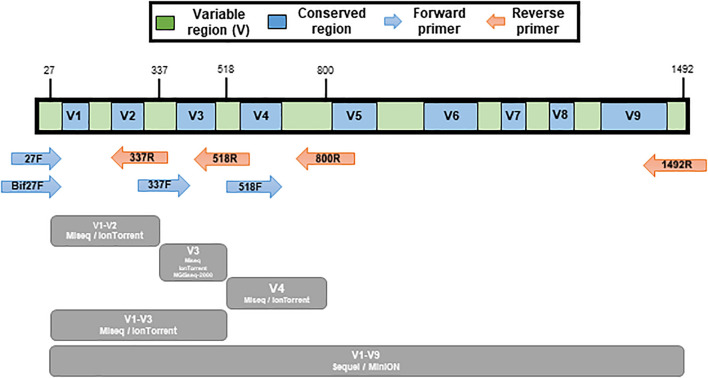
Illustration of variable regions within the 16S rRNA gene and the various primer pairs used for mock sample sequencing. Conserved regions are show in blue color and variable regions are green. Amplicon regions of each sequencing platform are shown in gray color.

The 16S rRNA gene PCR amplification of the mock communities was performed using universal primers covering variable regions of the 16S rRNA gene. The primers used in this study is listed in Table 2. The V1-V2 (27F/bif27F-337R), V3 (337F-518R), V4 (518F-800R), and V1-V3 (337F-800R) regions were amplified for the MiSeq (Illumina, United States) and IonTorrent (Thermo Fisher Scientific, United States). The V3 region was amplified for the MGIseq-2000 (MGI Tech, China), and the V1-V9 (27F/bif27F-1492R) region was amplified for MinION (Oxford Nanopore, United Kingdom) and Sequel II (Pacific Biosciences, United States) (Figure 2). The primers for MiSeq were universal primers with adapter sequence, and the primers for the other platform were universal primers without an additional sequence (Table 2). The amplifications were carried out under the following conditions: initial denaturation at 95°C for 1min, followed by 30 cycles of denaturation at 95°C for 15 s, primer annealing at 55°C for 15 s, and extension at 72°C for 90 s, with a final elongation at 72°C for 7 min using AccuPower^®^ Taq PCR PreMix & Master Mix (Bioneer, South Korea).

### 16S rRNA Gene Amplicon Sequencing

In the MiSeq platform, secondary amplification for attaching the NexTera sequence barcoding was performed with the i5 forward primer and i7 reverse primer (Table 2). The secondary primers consist of Illumina adapter, i5 or i7 index, and NexTera consensus sequences (Illumina, 2021). The amplified products were purified with CleanPCR (CleanNA, Netherlands). Equal concentrations of purified products were pooled together and short fragments (non-target products) were removed with CleanPCR. The quality and size of the sequencing libraries were assessed on a Bioanalyzer 2100 (Agilent, United States) using a DNA 7500 chip (Agilent, United States). The normalized sequencing libraries were pooled and sequencing was carried out at ChunLab, Inc., with the MiSeq Sequencing system, according to the manufacturer’s instructions.

In the IonTorrent platform, sequencing DNA libraries were constructed using the Ion Plus Fragment Library Kit (Thermo Fisher Scientific, United States) by following the manufacturer’s library preparation protocol for amplicons. The sequencing libraries were processed as barcoded libraries by using the Ion Code Barcode Adapter Kit. The barcoded libraries were subsequently quantified by qPCR using an Ion Universal Library Quantitation Kit (Thermo Fisher Scientific, United States). Reactions were performed as described by the manufacturer, and each library was diluted to a final concentration of 100 pmol. After individual quantitation, the barcoded libraries of each amplicon were pooled in equimolar amounts to ensure equal representation of each barcoded library in the sequencing run. Template preparation was performed by the Ion Chef System (Thermo Fisher Scientific, United States) using Ion 520^TM^ & Ion 530^TM^ ExT Kit-Chef and Sequencing kit (Thermo Fisher Scientific, United States) with the Ion 530 Chip (Thermo Fisher Scientific, United States) following the instructions of the manufacturers. All barcoded libraries were sequenced in single run.

In the MGIseq-2000 platform, gDNA (1 μg) was sheared using a S220 Ultra sonicator (Covaris, Canada). Sequencing library preparation was performed with an MGIEasy DNA library prep kit (BGI, China) according to the manufacturer’s instructions. Briefly, after size-selection of fragmented genomic DNA using AMPure XP magnetic beads (Beckman Coulter Genomics, United States), the fragmented genomic DNA was end-repaired and a-tailed at 37°C for 30 min and 65°C for 15 min. The indexing adapter was ligated to the ends of the DNA fragments at 23°C for 60 min. After the cleanup of adapter-ligated DNA, PCR was performed to enrich those DNA fragments that have adapter molecules. Thermocycler conditions were as follows: 95°C for 3 min, 7 cycles of 98°C for 20 s, 60°C for 15 s, and 72°C for 30 s, with a final extension at 72°C for 10 min. The indexed library was quantified using a QuantiFluor ONE dsDNA System (Promega, United States) and 330 ng in a total volume of 60 μl or less. The library was cyclized at 37°C for 60 min and then digested at 37°C for 30 min, followed by a cleanup of the circularization product. To make DNA nanoballs (DNBs), the library was incubated at 30°C for 25 min using the DNB enzyme. Finally, the library was quantified by a QuantiFluor ssDNA System (Promega, United States). The sequencing of the prepared DNBs was conducted on an MGIseq-2000 system (BGI, China) with 150 bp paired-end reads.

In the Sequel II platform, the PCR product was confirmed by using 1% agarose gel electrophoresis and visualized with a Gel Document system (Bio-Rad, United States). The amplified products were purified with AMpure PB beads (Pacific Biosciences, United States). Equal concentrations of purified products were pooled together and short fragments (non-target products) were removed with the AMpure PB beads. The quality and the size of the library were assessed on a Bioanalyzer 2100 (Agilent, United States) using a DNA 7500 chip (Agilent, United States). SMRTbell library construction and sequencing were carried out using the Pacific Biosciences sequel system (Pacific Biosciences, United States) according to the manufacturer’s instructions.

In the MinION platform, each PCR product (45 μl) was used for the end-prep process with dA-tailing, adding 7 μl of Ultra II End-prep reaction buffer (NEB, United States) and 3 μl of Ultra II End-prep enzyme mix (NEB, United States). After cleanup of end-prep DNA using AMPure XP beads (Beckman Coulter Genomics, United States), 22.5 μl of the end-prepped fragments were ligated with 25 μl Blunt/TA Ligase Master Mix and 2.5 μl of each of the barcode (NB01-NB08) of the native barcoding kit (SQK-LSK108). After cleanup of the barcode-ligated library using AMPure XP beads, an equimolar amount of pooled barcoded amplicons was mixed for adapter ligation with 50 μl of pooled template, 20 μl of native barcode adapter mix (BAM), 20 μl of NEBNext Quick Ligation Reaction Buffer (5×) (NEB, United States), and 10 μl of Quick T4 DNA Ligase. After cleanup of an adapter-ligated library, PCR was performed to enrich those DNA fragments that have adapter molecules. The prepared 12 μl of DNA library was mixed with 35 μl of RBF, 25.5 μl of LLB, and 2.5 μl of nuclease-free water for sample loading. The sequencing library DNA was loaded into a 1D-flow cell according to the manufacturer’s instructions and ran for 24 h. Raw data collection and base-calling was performed with MinKNOW software (version 19. 10. 1). The sequence reads used in this study was deposited in EBI Metagenomics (Accession number; PRJEB45207, ERP129281).

### Sequence Read Processing and Analysis

The MOTHUR (version 1.41.1) pipeline was used for the raw sequence data analyzing according to the MOTHUR SOP (Supplementary Figure 1; Schloss et al., 2009). All of the datasets were comprised 10,000 subsampled reads from each sample for comparative analysis. We then performed quality control, assembled contigs, aligned sequences, trimmed sequences, removed chimeras, classified sequences, calculated the error rate, and removed non 16S rRNA sequences of raw data (Supplementary Figure 1). Following the quality control step, raw data were simplified by the unique.seqs command. Sequences were aligned using the align.seqs command with EzBioCloud 16S database for MOTHUR (Chun et al., 2007; Kim et al., 2012; Yoon et al., 2017). The EzBioCloud database contains 66,303 rRNA gene sequences at species/phylotype and subspecies level. The database was manually curated and used as *de facto* standards for the taxonomical study of the prokaryotes (Hur et al., 2013; Kim et al., 2013, 2015, 2016, 2017; Chang et al., 2015; Lee et al., 2016; Zheng et al., 2016; Choi et al., 2017; Chun et al., 2018; Jang et al., 2020; Hugenholtz et al., 2021). The aligned sequence was trimmed using “screen.seqs” and the redundant reads were deleted using the “filter.seqs,” and “unique.seqs” commands. Chimeric sequences were determined using the “chimera.vsearch” command. The filtered reads were taxonomically assigned using the Eztaxon-e database for reference by the “classify.seqs” command. “make.shared” and “classify.otu” commands were used to calculate the taxonomic rank and the relative abundance for each phylotype. Rarefaction curves were produced using the “rarefaction.single” command. The “seq.error” command was used to calculate the sequencing error rate.

### Methods of Comparison Analysis

The taxonomical clustering of mock community samples was visualized with a heatmap created in shinyheatmap online^1^. The heatmap was clustered using Euclidean distance (Supplementary Figures 2–4). This program was run on R Shiny web server. The principal component analysis (PCA) of the microbial profiles was performed using Perseus (version 1.6.14.0).

The relative difference value of each strain was defined using the relative difference between the ratio of specific strains in the original community and in the sequenced results. The Bias Index (BI) value was introduced to compare bias between mock communities (Eq. 1). The value can be defined as the normalized Euclidean distance between the original mock community and sequenced mock community based on the relative difference in the ratio of each species. The positive relative difference values showed overrepresentation in the sequenced results while the negative value showed underrepresentation in the sequenced results. If the absolute value was higher than 0.5, it was regarded that the specific strain was greatly over-/underrepresented in the sequenced results. The BI converges to zero when the microbial profiles of the original mock community and sequenced mock community are similar. In contrast, a higher value of BI indicated higher bias.


$$B\,I=\frac{\sqrt{\sum_{i=1}^{N}{\left(\frac{{\bar{x}}_{i}-x_{i}}{x_{i}}\right)}^{2}}}{N-1}$$

Equation 1. Equation of Bias Index *N*: the number of strains in a mock community. *x_i_*: The original ratio of individual stains, ${\bar{x}}_{i}$: the ratio of sequenced individual strains.

## Results

### The Quantification of Genomic DNA

Each genomic DNA of the strains was quantified using a Quantus Fluorometer and ddPCR in triplicated. The concentrations of *L. acidophilus*, *L. fermentum*, *L. gasseri*, *L. paracasei* subsp. *paracasei*, *L. reuteri*, *B. animalis* subsp. *lactis*, *B. breve*, and *L*. *lactis* are 1.16, 1.27, 1.49, 1.4, 1.36, 1.51, 1.63, and 1.87 per 1 ng/μl, respectively. The standard deviation (SD) of quantified gDNA was calculated (Supplementary Table 2). The concentration of each stain is summarized in Supplementary Table 2. After measuring the concentration, the template was serially diluted and used for the determination of copy number by ddPCR. The average copy numbers of *L. acidophilus*, *L. fermentum*, *L. gasseri*, *L. paracasei* subsp. *paracasei*, *L. reuteri*, *B. animalis* subsp. *lactis*, *B. breve*, and *L*. *lactis* are 6.7 × 10^6^, 6.5 × 10^6^, 2.3 × 10^7^, 5.2 × 10^6^, 3.8 × 10^6^, 6.9 × 10^6^, 4.9 × 10^6^, and 2.8 × 10^6^ per 1 μl, respectively (Supplementary Table 2).

The ratio of each genomic DNA in a mock community was determined by the copy number of 16S rRNA genes (Supplementary Tables 2, 3). The sample in mock A consists of *L. acidophilus* (18.6%), *L. fermentum* (18.45%), *L. gasseri* (17.48%), *L. paracasei* subsp. *paracasei* (20.18%), and *L. reuteri* (25.3%). In mock B, it consists of *B. animalis* subsp. *lactis* (46.55%) and *B. breve* (53.45%). In the mock C, it consists of *L. acidophilus* (16.62%), *L. fermentum* (16.79%), *L. gasseri* (15.98%), *L. paracasei* subsp. *paracasei* (18.16%), *L. reuteri* (15.32%), and *L*. *lactis* (17.13%). In mock D, it consists of *B. animalis* subsp. *lactis* (30.73%), *B. breve* (35.14%), and *L*. *lactis* (34.13%). In mock E consists of *L. acidophilus* (33.41%), *L. fermentum* (26.73%), *L. gasseri* (19.12%), *L. paracasei* subsp. *paracasei* (14.57%), and *L. reuteri* (6.16%). In mock F, it consists of *B. animalis* subsp. *lactis* (56.64%) and *B. breve* (43.36%). In the mock G, it consists of *L. acidophilus* (8.11%), *L. fermentum* (8.08%), *L. gasseri* (7.78%), *L. paracasei* subsp. *paracasei* (8.88%), *L. reuteri* (7.53%), *B. animalis* subsp. *lactis* (16.99%), *B. breve* (19.43%), and *L*. *lactis* (23.21%). In mock H, it consists of *L. acidophilus* (12.56%), *L. fermentum* (12.51%), *L. gasseri* (11.96%), *L. paracasei* subsp. *paracasei* (13.63%), *L. reuteri* (11.58%), *B. animalis* subsp. *lactis* (11.55%), *B. breve* (13.3%), and *L*. *lactis* (12.91%).

### Sequence Statistics

The input reads were subsampled from the total reads to 10,000 reads. The redundant, chimeric, reads after non-bacteria 16S rRNA sequences were trimmed in the MOTHUR pipeline. The MiSeq platform showed 70,876 (mean) ± 19,543 (SD) for V1-V2, 75,451 ± 18,013 for V3, 75,805 ± 18,028 for V4, and 56,793 ± 19,029 for V1-V3 and for the total dataset 69,731 ± 19,939 reads per sample, respectively. The average accuracy of this platform is 99.720%. The IonTorrent platform showed 130,742 (mean) ± 27,042 (SD) for V1-V2, 213,401 ± 45,200 for V3, 140,429 ± 16,299 for V4, 126,217 ± 13,493 for V1-V3, and for the total dataset 152,697 ± 45,254 reads per sample, respectively. The average accuracy of this platform is 99.825%. The MGIseq-2000 produced 1,379,653 (mean) ± 484,553 (SD) V3 reads per sample. The average accuracy of this platform is 99.996%. From the amplicon dataset, Sequel II produced 20,556 (mean) ± 5,034 (SD) V1-V9 reads per sample, respectively. The average accuracy of this platform is 99.708%. The MinION platform showed 81,000 (mean) ± 32726.14 (SD) for V1-V9 reads per sample, respectively. The average accuracy of this platform is almost 99.453%. Supplementary Table 4 summarizes the read number through the trimming process.

### Analysis by Next-Generation Sequencing Platforms Based on Short-Read

#### MiSeq

The sequencing of mock communities using MiSeq was done with four primer pairs in triplicate. The primer pairs targeted different variable regions: V1-V2, V3, V4, and V1-V3 (Figure 2 and Table 2).

The sequencing results of mock A showed that *L. acidophilus* was greatly underrepresented compared to the ratio of *L. acidophilus* (18.6%) in the original mock community. The sequence read ratios (SRR) of *L. acidophilus* were 8.83% (V1-V2), 8.64% (V3), 7.76% (V4), and 6.59% (V1-V3). In contrast, the SRR of *L. gasseri* were greatly overrepresented compared to the ratio of *L. gasseri* (17.48%) in the original mock community. The SRRs of *L. gasseri* were 26.43% (V1-V2), 22.87% (V3), 29.29% (V4), and 27.71% (V1-V3). *L. reuteri* is relatively well represented in all sequencing regions.

The sequencing results of mock B showed that *B. animalis* subsp. *lactis* was overrepresented compared to the ratio of *B. animalis* subsp. *lactis* (46.55%) in the original mock community. The SRRs of *B. animalis* subsp. *lactis* were 53.55% (V1-V2), 55.15% (V3), 74.1% (V4), and 84.21% (V1-V3). In contrast, the SRRs of *B. breve* were underrepresented compared to the ratio of *B. breve* (53.45%) in the original mock community. The SRRs of *B. breve* were 46.45% (V1-V2), 44.85% (V3), 25.9% (V4), and 15.79% (V1-V3).

The sequencing results of mock C showed that *L. acidophilus* was greatly underrepresented compared to the ratio of *L. acidophilus* (16.62%) in the original mock community. The SRRs of *L. acidophilus* were 5.54% (V1-V2), 5.29% (V3), 4.8% (V4), and 2.26% (V1-V3). In contrast, the SRRs of *L*. *lactis* were greatly overrepresented compared to the ratio of *L*. *lactis* (17.13%) in the original mock community. The SRRs of *L*. *lactis* were 27.46% (V1-V2), 27.5% (V3), 31.61% (V4), and 40.88% (V1-V3). *L. reuteri* was relatively well represented in all sequencing regions.

The sequencing results of mock D showed that *B. breve* was underrepresented compared to the ratio of *B. breve* (35.14%) in the original mock community. The SRRs of *B. breve* were 29.35% (V1-V2), 20.88% (V3), 15.57% (V4), and 9.16% (V1-V3). In contrast, the SRRs of *L*. *lactis* were overrepresented compared to the ratio of *L*. *lactis* (34.13%) in the original mock community. The SRRs of *L*. *lactis* were 35.61% (V1-V2), 49.88% (V3), 53.05% (V4), and 52.74% (V1-V3). However, *B. animalis* subsp. *lactis* was well represented relative to the original mock community.

The sequencing results of mock E showed that *L. acidophilus* was greatly underrepresented compared to the ratio of *L. acidophilus* (33.41%) in the original mock community. The SRRs of *L. acidophilus* were 15.66% (V1-V2), 15.46% (V3), 14.85% (V4), and 7.32% (V1-V3). In contrast, the SRRs of *L. reuteri* were greatly overrepresented compared to the ratio of *L. reuteri* (6.16%) in the original mock community. The SRRs of *L. reuteri* were 9.14% (V1-V2), 10.11% (V3), 9.93% (V4), and 7.11% (V1-V3). However, *L. gasseri* was well represented relative to the original mock community in the V1-V2, V3, and V4 regions.

The sequencing results of mock F showed that *B. animalis* subsp. *lactis* was overrepresented compared to the ratio of *B. animalis* subsp. *lactis* (56.64%) in the original mock community. The SRRs of *B. animalis* subsp. *lactis* were 61.74% (V1-V2), 63.12% (V3), 75.91% (V4), and 85.23% (V1-V3). In contrast, the SRRs of *B. breve* were underrepresented compared to the ratio of *B. breve* (43.36%) in the original mock community. The SRRs of *B. breve* were 38.26% (V1-V2), 36.88% (V3), 24.09% (V4), and 14.77% (V1-V3).

The sequencing results of mock G showed that *L. acidophilus* was greatly underrepresented compared to the ratio of *L. acidophilus* (8.11%) in the original mock community. The SRRs of *L. acidophilus* were 1.89% (V1-V2), 2.74% (V3), 2.87% (V4), and 1.14% (V1-V3). In contrast, the SRR of *L*. *lactis* were overrepresented compared to the ratio of *L*. *lactis* (23.21%) in the original mock community. The SRRs of *L*. *lactis* were 23.59% (V1-V2), 32.13% (V3), 34.56% (V4), and 35.35% (V1-V3). However, *L. fermentum* was well represented relative to the original mock community.

The sequencing results of mock H showed that *L. acidophilus* was greatly underrepresented compared to the ratio of *L. acidophilus* (12.56%) in the original mock community. The SRRs of *L. acidophilus* were 3.08% (V1-V2), 3.96% (V3), 4.06% (V4), and 1.33% (V1-V3). In contrast, the SRRs of *L*. *lactis* were greatly overrepresented compared to the ratio of *L*. textitlactis (12.91%) in the original mock community. The SRRs of *L*. *lactis* were 15.12% (V1-V2), 21.96% (V3), 23.81% (V4), and 27.46% (V1-V3). However, *L. reuteri* was well represented relative to the original mock community.

**FIGURE 3:**
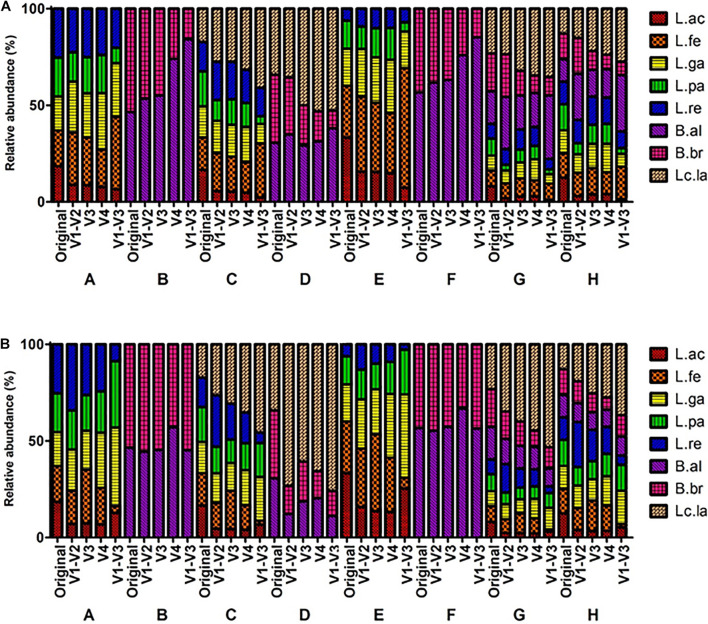
Comparison of mock communities’ microbial profiles based on the sequencing reads from MiSeq and IonTorrent platform. V1-V2, V3, V4, and V1-V3 indicated the amplified variable region of the amplicon. A–H indicated specific mock communities. **(A)** MiSeq Sequencing profiling of relative abundance on mock communities. **(B)** IonTorrent Sequencing profiling of relative abundance on mock communities.

**FIGURE 4:**
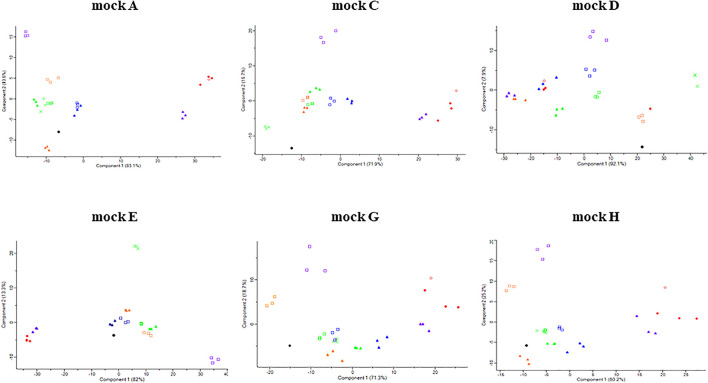
Principle component analysis (PCA) of original and sequenced mock communities. The symbols are based on the following criteria: ●, original; □, MiSeq; ▲, IonTorrent; X, MGIseq-2000; ◆, Sequel II; and ◇, MinION. The orange, green, blue, violet, and red color indicate V1-V2, V3, V4, V1-V3, and V1-V9 regions, respectively.

The microbial profiles from MiSeq platform showed that *L. acidophilus* was underrepresented while *L*. *lactis* was overrepresented throughout all mock communities. The BI value of the V1-V3 region was relatively higher than that of other regions throughout all mock communities (Supplementary Table 5), indicating the V1-V3 regions had the greatest bias. This was also supported by the heatmap and PCA. In the heatmap analysis, the profiles of the V1-V3 region formed a distinguished group in the phylogenetic tree while the profiles of the original and other regions formed a monophyletic group (Figure 3A and Supplementary Figure 2). These clusters were also observed in the PCA results (Figure 4).

#### IonTorrent

The sequencing of mock communities using IonTorrent was done with four primer pairs in triplicate. The primer pairs targeted different variable regions: V1-V2, V3, V4, and V1-V3 (Figure 2 and Table 2).

The sequencing results of mock A showed that *L. acidophilus* was greatly underrepresented compared to the ratio of *L. acidophilus* (18.6%) in the original mock community. The SRRs of *L. acidophilus* were 7.12% (V1-V2), 7.38% (V3), 6.82% (V4), and 12.78% (V1-V3). In contrast, the SRR of *L. gasseri* were greatly overrepresented compared to the ratio of *L. gasseri* (17.48%) in the original mock community. The SRRs of *L. gasseri* were 21.18% (V1-V2), 20.21% (V3), 28.5% (V4), and 40.74% (V1-V3). In the V1-V3 region, *L. fermentum* and *L. reuteri* were underrepresented compared to the original mock community.

The V4 region sequencing results of mock B showed that *B. animalis* subsp. *lactis* was overrepresented compared to the ratio of *B. animalis* subsp. *lactis* (46.55%) in the original mock community. The SRR of *B. animalis* subsp. *lactis* was 57.06% (V4). In contrast, the SRR of *B. breve* was underrepresented compared to the ratio of *B. breve* (53.45%) in the original mock community. The SRR of *B. breve* was 42.94% (V4). However, the strains of V1-V2, V3, and V4 regions were well represented relative to the original mock community.

The sequencing results of mock C showed that *L. acidophilus* was greatly underrepresented compared to the ratio of *L. acidophilus* (16.62%) in the original mock community. The SRRs of *L. acidophilus* were 4.57% (V1-V2), 4.42% (V3), 4.1% (V4), and 6.72% (V1-V3). In contrast, the SRRs of *L*. *lactis* were greatly overrepresented compared to the ratio of *L*. *lactis* (17.13%) in the original mock community. The SRRs of *L*. *lactis* were 26.32% (V1-V2), 30.64% (V3), 35.2% (V4), and 45.57% (V1-V3). *L. gasseri* was relatively well represented in all sequencing regions.

The sequencing results of mock D showed that *B. animalis* subsp. *lactis* was underrepresented compared to the ratio of *B. animalis* subsp. *lactis* (30.73%) in the original mock community. The SRRs of *B. animalis* subsp. *lactis* were 12.25% (V1-V2), 18.8% (V3), 20.46% (V4), and 11.23% (V1-V3). In contrast, the SRRs of *L*. *lactis* were greatly overrepresented compared to the ratio of *L*. *lactis* (34.13%) in the original mock community. The SRRs of *L*. *lactis* were 73.26% (V1-V2), 60.74% (V3), 65.64% (V4), and 75.93% (V1-V3).

The sequencing results of mock E showed that *L. acidophilus* was greatly underrepresented compared to the ratio of *L. acidophilus* (33.41%) in the original mock community. The SRRs of *L. acidophilus* were 15.88% (V1-V2), 13.63% (V3), 13.13% (V4), and 25.43% (V1-V3). In contrast, the SRRs of *L. gasseri* were overrepresented compared to the ratio of *L. gasseri* (19.12%) in the original mock community. The SRRs of *L. gasseri* were 25.51% (V1-V2), 23.28% (V3), 33.03% (V4), and 43.12% (V1-V3).

The sequencing results of mock F showed that *B. animalis* subsp. *lactis* was overrepresented compared to the ratio of *B. animalis* subsp. *lactis* (56.64%) in the original mock community. The SRRs of *B. animalis* subsp. *lactis* were 55.27% (V1-V2), 57.32% (V3), 66.81% (V4), and 56.51% (V1-V3). In contrast, the SRRs of *B. breve* were underrepresented compared to the ratio of *B. breve* (43.36%) in the original mock community. The SRRs of *B. breve* were 44.73% (V1-V2), 42.68% (V3), 33.19% (V4), and 43.49% (V1-V3).

The sequencing results of mock G showed that *L. acidophilus* was greatly underrepresented compared to the ratio of *L. acidophilus* (8.11%) in the original mock community. The SRRs of *L. acidophilus* were 2.36% (V1-V2), 2.31% (V3), 2.2% (V4), and 3.14% (V1-V3). In contrast, the SRR of *L*. *lactis* were greatly overrepresented compared to the ratio of *L*. *lactis* (23.21%) in the original mock community. The SRRs of *L*. *lactis* were 34.66% (V1-V2), 39.92% (V3), 44.59% (V4), and 53.23% (V1-V3).

The sequencing results of mock H showed that *L. acidophilus* was greatly underrepresented compared to the ratio of *L. acidophilus* (12.56%) in the original mock community. The SRRs of *L. acidophilus* were 3.96% (V1-V2), 3.45% (V3), 3.55% (V4), and 5.32% (V1-V3). In contrast, the SRRs of *L*. *lactis* were greatly overrepresented compared to the ratio of *L*. *lactis* (12.91%) in the original mock community. The SRRs of *L*. *lactis* were 19.13% (V1-V2), 25.31% (V3), 27.43% (V4), and 36.62% (V1-V3). In the V1-V3 region, *L. fermentum* and *L. reuteri* were greatly underrepresented compared to the original.

**FIGURE 5:**
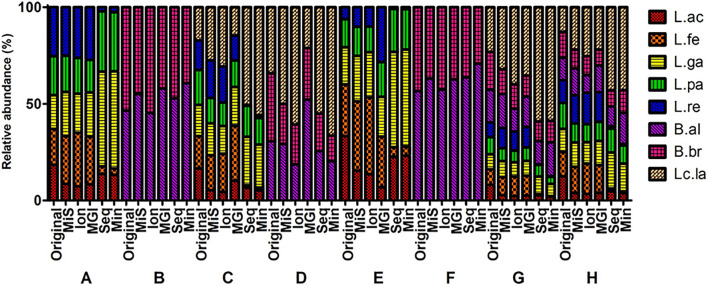
Comparison of V3 region sequencing analysis of mock communities by five NGS platforms. Original, MiS: MiSeq, Ion: IonTorrent, MGI: MGIseq-2000, Seq: Sequel, Min: MinION. A–H indicates specific mock communities.

As a result, from the analysis using the IonTorrent platform, *L. acidophilus* was underrepresented while *L*. *lactis* was overrepresented. However, the mock B and D containing *Bifidobacterium* species were relatively well represented compared to MiSeq (Figure 5 and Supplementary Table 5). The BI value of the V1-V3 region was relatively higher than that of other regions throughout all mock communities (Supplementary Table 5), indicating the V1-V3 regions had the greatest bias. This also was supported by the heatmap and PCA. In the heatmap analysis, the profiles of the V1-V3 region formed a distinguished group in the phylogenetic tree while the profiles of the original and other regions formed a monophyletic group (Figure 3B and Supplementary Figure 3). These clusters were also observed in the PCA results (Figure 4).

#### MGIseq-2000

The sequencing of mock communities using MGIseq-2000 was done with the primer pair that targeted the variable V3 region in triplicate (Figure 2 and Table 2).

The sequencing results of *L. acidophilus* were greatly underrepresented compared to the ratios of *L. acidophilus* 18.6% (A), 16.62% (C), 33.41% (E), 8.11% (G), and 12.56% (H) in the original mock community. The SRRs of the mock communities were 8.35% (A), 10.25% (C), 6.83% (E), 2.57% (G), and 3.98% (H). In contrast, the SRR of *L. reuteri* was greatly overrepresented compared to the ratios of *L. reuteri* 25.3% (A), 6.16% (E), 7.53% (G), and 11.58% (H) in the original mock community. The SRRs of mock communities were 27.27% (A), 28.37% (E), 10.6% (G), and 15.36% (H). In some mock communities (mock A, G, and H) the results of MGIseq2000 were clustered with the results from V3 region sequencing using MiSeq and IonTorrent (Supplementary Figure 4). However, the results from MGIseq-2000 were distinguished from the results using MiSeq and IonTorrent, especially mock E. The BI value of mock E was relatively higher than that of other regions throughout all mock communities (Supplementary Table 5), indicating mock E had the greatest bias. This also supported by heatmap and PCA. In heatmap analysis, the profiles of mock E formed a distinguished group in the phylogenetic tree while the profiles of original and other regions formed a monophyletic group (Supplementary Figure 4). These clusters were also observed in the PCA results (Figure 4).

### Analysis by Next-Generation Sequencing Platforms Based on Long-Read

#### Sequel II

The sequencing of mock communities using Sequel II was done with the primer pair that targeted the variable V1-V9 region in triplicate (Figure 2 and Table 2).

The sequencing results of *L. fermentum* was greatly underrepresented compared to the ratios of *L. fermentum* 18.45% (A), 16.79% (C), 26.73% (E), 8.08% (G), and 12.51% (H) in the original mock community. The SRRs of mock communities were 3.96% (A), 1.41% (C), 5.06% (E), 0.62% (G), and 1.4% (H). In contrast, the SRRs of *L*. *lactis* were greatly underrepresented compared to the ratio of *L*. *lactis* 17.13% (C), 34.13% (D), 23.21% (G), and 12.91% (H) in the original mock community. The SRRs of mock communities were 50.57% (C), 55.03% (D), 59.05% (G), and 43% (H). In the PCA results, the points of the mock communities excluding mock B and F were separated from those of other samples (Figure 4). The BI value of mock A was relatively higher than that of other regions throughout all mock communities (Supplementary Table 5), indicating mock A had the greatest bias. This was also supported by the heatmap and PCA results. In the heatmap analysis, the profiles of one mock E sample formed a distinguished group in the phylogenetic tree while the profiles of the original and other regions formed a monophyletic group (Supplementary Figure 4). These clusters were also observed in the PCA results (Figure 4).

#### MinION

The sequencing of mock communities using MinION was done with the primer pair that targeted the variable V1-V9 region (Figure 2 and Table 2).

The sequencing results of *L. fermentum* were greatly underrepresented compared to the ratios of *L. fermentum* 18.45% (A), 16.79% (C), 26.73% (E), 8.08% (G), and 12.51% (H) in the original mock community. The SRRs of the mock communities were 3.27% (A), 1.51% (C), 4.71% (E), 0.37% (G), and 0.9% (H). In contrast, the SRR of *L*. *lactis* were greatly underrepresented compared to the ratios of *L*. *lactis* 17.13% (C), 34.13% (D), 23.21% (G), and 12.91% (H) in the original mock community. The SRRs of mock communities were 55.8% (C), 66.35% (D), 58.75% (G), and 42.97% (H). In the PCA results, the points of the mock communities excluding mock B, D, and F were separated from those of other samples (Figure 4). The BI value of mock A was relatively higher than that of other regions throughout all mock communities (Supplementary Table 5), indicating mock A had the greatest bias. This was also supported by the heatmap and PCA results. In the heatmap analysis, the profiles of eight samples formed a distinguished group in the phylogenetic tree while the profiles of the original and other platforms samples formed a monophyletic group (Supplementary Figure 4). These clusters were also observed in the PCA results (Figure 4).

### Comparison of Five Platforms Reads Analysis

The V3 region data of five NGS platforms were compared (Figure 5). The MiSeq, IonTorrent, and MGIseq-2000 platforms have higher accuracy than the Sequel II and MinION platforms in mock A, E, G, and H. The MiSeq and Sequel II platforms have high accuracy in mock B. The MGIseq-2000 platform has high accuracy in mock C and D. The IonTorrent platform has high accuracy in mock F. As a result, the MiSeq and MGIseq-2000 platforms based on paired-end sequencing technology were more accurate than single read sequencing platforms. On the other hand, data from the Sequel II and MinION platforms based on long-read sequencing technologies were highly biased in mock A, C, E, G, and H. In all NGS platforms, *L. acidophilus* was greatly underrepresented while *L*. *lactis* was generally overrepresented. In the Sequel II and MinION platforms, *L. fermentum* and *L. reuteri* were highly underrepresented compared to the original composition.

## Discussion

Developments in NGS technology allow microbiome analyses to be carried out for various environments. They also allow microbiome analysis of the human gastrointestinal, oral, skin, nasal, and vaginal regions and other organs in the body (Fettweis et al., 2012). There are a variety of PCR methods, NGS library kits, NGS platforms, and microbiome analysis tools for the 16S rRNA gene that can affect sequencing bias (Poretsky et al., 2014; Boers et al., 2019). In this study, we compared the bias due to differences in five NGS platforms and each library kit that may affect the sequencing results. In this study, eight genomic DNAs of probiotic strains were initially quantified using a Quantus Fluorometer and ddPCR. The differences between the two instrument values can be harmonized using a reference material. The ddPCR can provide absolute quantification of the DNA amount by counting the number of positive amplification reactions from each DNA molecule, without standard curves or calibration, as required in quantitative PCR (White et al., 2009). The quantification based on the fluorometer measures the total amount of nucleic acids rather than the amount of specific genes. However, the saturation of droplets can be crucial for the ddPCR. Droplets with a high level indicate that a single droplet is likely to have more than one template. Although the saturation problem can be adjusted based on a Poisson distribution, the dynamic range of the ddPCR measurement is relatively narrow. In one study, the authors experimentally demonstrated that this ddPCR can achieve a linear dynamic range of four times magnitude (20,000-droplets analysis) for DNA quantification (Hindson et al., 2011; Pinheiro et al., 2012). The results were within dynamic ranges, according to the manufacturer’s technical document (Bio-Rad laboratories, Inc., 2015). A small amount of positive droplet was also detected in the negative control (NTC) sample. However, the remnant bacterial genomic DNA from recombinant Taq polymerase can be amplified with a universal bacterial primer without an additional template (Harinder and Makkar, 2005).

In NGS platforms based on short-reads, several strains were classified as different taxons by MOTHUR. The strain of *L. paracasei* subsp. *paracasei* was classified as *L. zeae*, and *B. animalis* subsp. *lactis* as *B. pseudolongum* in the V3 region. The strain of *L. fermentum* is classified as GQ156395s, *L. gasseri* as CP006809s, *L. paracasei* subsp. *paracasei* as *L. zeae*, *L. reuteri* as *L. panis*, and *B. breve* as *B. longum* in the V4 region. However, the classified taxon sequence had the same sequence as the mock strain in the V3 or V4 region. *L. paracasei* and *L. zeae* are members of the *Lactobacillus casei* group (LCG) and LCG composed of very closely related *Lactobacillus* species (Hill et al., 2018). It is also known that identification of LCG with 16S rRNA gene sequencing is difficult (Huang et al., 2018). These findings indicate that 16S rRNA gene sequencing with the V3 and V4 regions should not be recommended for the identification of probiotic bacteria.

The BI of the sequenced profile ratio showed specific species dependent bias (Supplementary Table 5). In, the MiSeq platform, the V1-V3 region of all mock communities has a higher BI value than that of other regions (Supplementary Table 5). The underrepresented *L. acidophilus* and the overrepresented *L*. *lactis* had a major contribution to this bias. In both the MiSeq and IonTorrent platforms, the V1-V3 region of all mock communities has a higher BI value than those of other regions excepting mock B and F with IonTorrent. In all mock communities, the V1-V3 region point was clearly separated from the original and other regions. The PCA and heatmap analysis results showed that most microbial profiles from short reads except V1-V3 regions were clustered with the original profile (Figure 4 and Supplementary Table 5). The higher BI value and bias of the V1-V3 regions might originate from longer amplicon size compared to other regions (Table 3). Combining low nucleotide diversity of the amplicon sequencing, the quality of individual reads can be decreased, especially at the end of individual reads.

**TABLE 3:** The information of GC contents and amplicon length in each sequencing region.

Information	V1-V2		V3		V4		V1-V3		V1-V9	
strains	GC (%)	Length (bp)	GC (%)	Length (bp)	GC (%)	Length (bp)	GC (%)	Length (bp)	GC (%)	Length (bp)
*Lactobacillus acidophilus*	54.8	332	46.3	160	52.2	247	52.5	512	54	1,528
*Limosilactobacillus fermentum*	52.5	343	46.9	160	50.6	247	51.2	523	53	1,540
*Lactobacillus gasseri*	50.7	339	46.3	160	51.4	247	49.9	519	52	1,537
*Lacticaseibacillus paracasei* subsp. *paracasei*	51.9	335	51.9	160	51.8	247	52.4	515	53	1,534
*Limosilactobacillus reuteri*	51.3	339	51.9	160	53.8	247	52	523	54	1,540
*Bifidobacterium animalis* subsp. *Lactis*	61.3	310	56.6	152	59.9	247	60	482	60	1,502
*Bifidobacterium breve*	61.4	308	53.1	145	58.7	247	59	473	59	1,495
*Lactococcus lactis* subsp. *Lactis*	49.4	316	49.7	161	51.2	246	50.1	497	52	1,526

The MiSeq (Illumina) and IonTorrent (Thermo Fisher) platforms showed that the V1-V2 region sequencing yielded the lowest bias. However, it was reported that the results of amplicon-based study can be biased by amplicon size, GC content, and PCR artifacts. A previous study demonstrated that even with longer variable regions, more advanced sequencing techniques, and a wider range, it is clear that the microbial diversity measured in the same sample varies greatly with the choice of variable regions. Although 337F primer was used widely as universal 16S rRNA gene primer, There was one mismatch with *Lactobacillus* strains in the end of 5′ termini. This might affected the bias of V3-V4 region during PCR.

In this study, the genomic DNA was quantified using the EvaGreen system with the universal primer alone. Although the primer pairs were specific to the 16S rRNA gene sequence, the EvaGreen system measures the whole DNA contents of the PCR reaction. This indicates that some bias can arise from PCR artifacts and residual non-target DNA. This bias might be reduced with the Taqman probe system. The Taqman probe system can show higher relative sensitivity and dynamic range than the EvaGreen system, although additional Taqman probes are required for the assay (Fernandes et al., 2018). It was also reported the amplification efficiency can be interfered with by genomic DNA as a matrix in the mock community (Bonk et al., 2018).

In this study, *L. acidophilus* and *L*. *lactis* showed high biases regardless of the sequencing platforms. In a previous study, it was suggested that high genomic GC content can interfere with PCR reactions (Laursen et al., 2017). However, the relationship between high genomic GC content and bias was not confirmed in this study (Tables 1, 3). Another study showed that the low-GC regions can affect the bias of sequencing profiling (Sato et al., 2019). In this study, the genomes of *L. acidophilus* and *L*. *lactis* contained low genomic GC content (approximately 35%) and showed greatly underrepresentation or overrepresentation. It was unclear whether the genomic contents of the strain affect the PCR reaction, although some studies showed that low GC content can affect the bias of library preparations (Sato et al., 2019; Li et al., 2020).

Eight probiotics strains were selected based on the phylogenetic distance among nineteen listed probiotics of Ministry of Food and Drug Safety in KOREA. The probiotics bacteria are not generally classified as gut or fecal microbes. According to Claesson et al. (2010), they showed that significant bias compared to the other regions. The results of Chen et al. (2019), they demonstrated that the V3-V4 region showed similar results like V1-V2 and V4. Also, the V3-V4 region primers showed the highest abundance of specific strain. However, the composition and ratio of the mock community were known and absolutely quantified by ddPCR in this study. The strains used in this studies were probiotics strains and there can be difference in bias compared to human-gut or environmental microbial communities.

Though full-length 16S rRNA gene sequencing can provide the highest resolution for sequence identification (Myer et al., 2016), the long-read sequencing platforms of Sequel II and MinION showed higher bias than short read sequencing platforms in our study. The PCA results showed that the results of full-length sequences were relatively well clustered together, indicating that the bias mainly originated from PCR reaction bias rather than the sequencing preference of the long read sequencers. A previous study showed that different PCR thermal cycling conditions can interfere with the results with the same primer pairs and sequencing instruments, although the dissimilarity value of each thermal condition was similar (Fujiyoshi et al., 2020). In Figure 4, the PCA plot of mock B and F were not drawn as there are only two strains in the mock B and F. Those mock communities can be visually compared with the original in Figures 3, 5. In addition, the BI value was summarized as a matrix in Supplementary Table 5.

The bias can also arise from the bioinformatics process, although the effects are relatively minor. One study compared three 16S rRNA gene bioinformatics tools, QIIME, MOTHUR, and MG-RAST, using intestinal microbial composition data collected from preterm infants (Plummer and Twin, 2015). They measured the microbiome composition of the sample using three pipelines and found that there was little difference in *firmicutes* and *actinobacteria* phyla, and only a difference in terms of the time required to analyze the sample and convenient use was observed. In this study, the biases generated by 16S rRNA variable regions or NGS platforms were assessed. Our results indicated that the sequence-dependent bias is the most dominant factor of the bias. Though some difference in bias was observed regarding to the sequencing instruments, over- or underrepresentation of specific species was observed throughout all sequencing instruments or amplified variable regions. These biases can also originate from or be amplified by DNA extraction or PCR reaction (Boers et al., 2019). According to Callahan et al. (2016), MOTHUR and QIIME can be spurious output like chimeric and non-chimeric errors. However, redundant reads, chimeric sequence, and non 16S rRNA sequence were removed during MOTHUR procedure. Also, our study focused on comparison study of known microbiome composition rather than microbiome diversity study.

McLaren et al. (2019) suggested that the bias of metagenomics analysis can be fully described as the relative efficiencies of the whole process. This indicates that the metagenomics bias can be corrected if the relative efficiency can be measured. This relative efficiency or bias factor can be measured with the standards. Sequins is one of the standards for metagenomics (Hardwick et al., 2018). Sequins is a set of sequencing spike-in standards that mirrors sequences of natural genomic sequences. Due to the reversed sequences of sequins, sequins can be used as internal standards for metagenomics analysis. However, sequins is a set of synthetic genome sequences and it may not be possible to make sequins of every bacterial strain. Our study showed that the well-quantified mock community can be used assess the bias from different procedure, suggesting a well-quantified mixture of genomic materials or bacterial cells can be used as good standards for metagenomics study. As PCR is one of the outstanding methods for accurate and absolute quantification of specific genes, a mock community quantified with digital PCR might be a good standard for metagenomics study.

## Data Availability Statement

The datasets presented in this study can be found in online repositories. The names of the repository/repositories and accession number(s) can be found below: https://www.ebi.ac.uk/ena/browser/view/PRJEB45207.

## Author Contributions

CP and SK: conception and design. CP: performed the experiments, data analysis, and writing of the draft manuscript. CP, SBK, SC, and SK: review and editing the manuscript. All authors contributed to the article and approved the final version of the manuscript.

## Conflict of Interest

The authors declare that the research was conducted in the absence of any commercial or financial relationships that could be construed as a potential conflict of interest.

## Publisher’s Note

All claims expressed in this article are solely those of the authors and do not necessarily represent those of their affiliated organizations, or those of the publisher, the editors and the reviewers. Any product that may be evaluated in this article, or claim that may be made by its manufacturer, is not guaranteed or endorsed by the publisher.

## Footnotes

1
http://shinyheatmap.com
