# Supplementary material for: Comparison of 16S rRNA Gene Based Microbial Profiling Using Five Next-Generation Sequencers and Various Primers
Source: Front Microbiol. 2021 Oct 14;12:715500. doi: 10.3389/fmicb.2021.715500 (PMC8552068; doi:10.3389/fmicb.2021.715500)
Supplement: Supplementary file 1 [file Presentation_1.PPTX]

## Slide 1
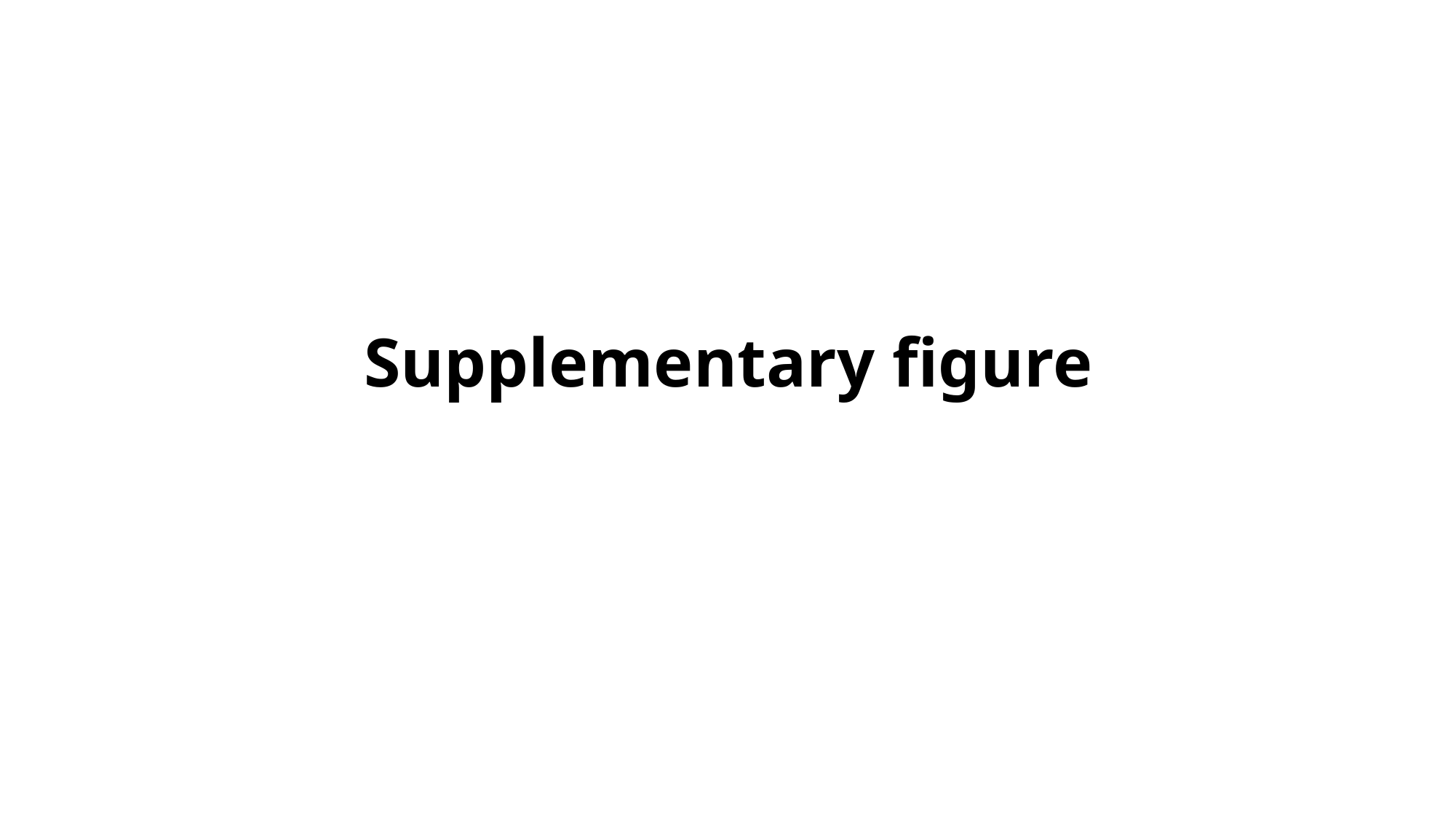

# Supplementary figure

## Slide 2
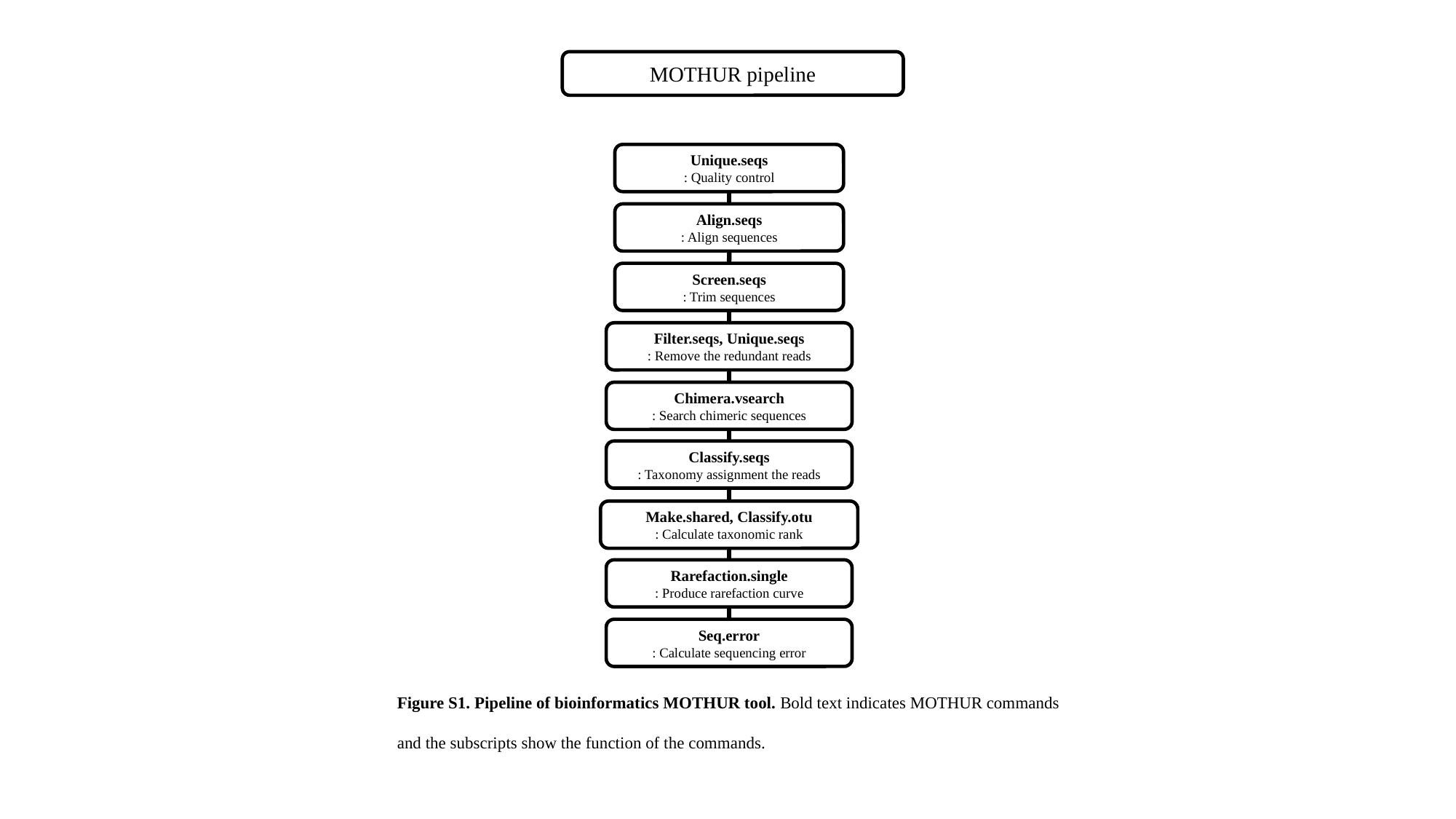

MOTHUR pipeline
Unique.seqs
: Quality control
Align.seqs
: Align sequences
Screen.seqs
: Trim sequences
Filter.seqs, Unique.seqs
: Remove the redundant reads
Chimera.vsearch
: Search chimeric sequences
Classify.seqs
: Taxonomy assignment the reads
Make.shared, Classify.otu
: Calculate taxonomic rank
Rarefaction.single
: Produce rarefaction curve
Seq.error
: Calculate sequencing error
Figure S1. Pipeline of bioinformatics MOTHUR tool. Bold text indicates MOTHUR commands and the subscripts show the function of the commands.

## Slide 3
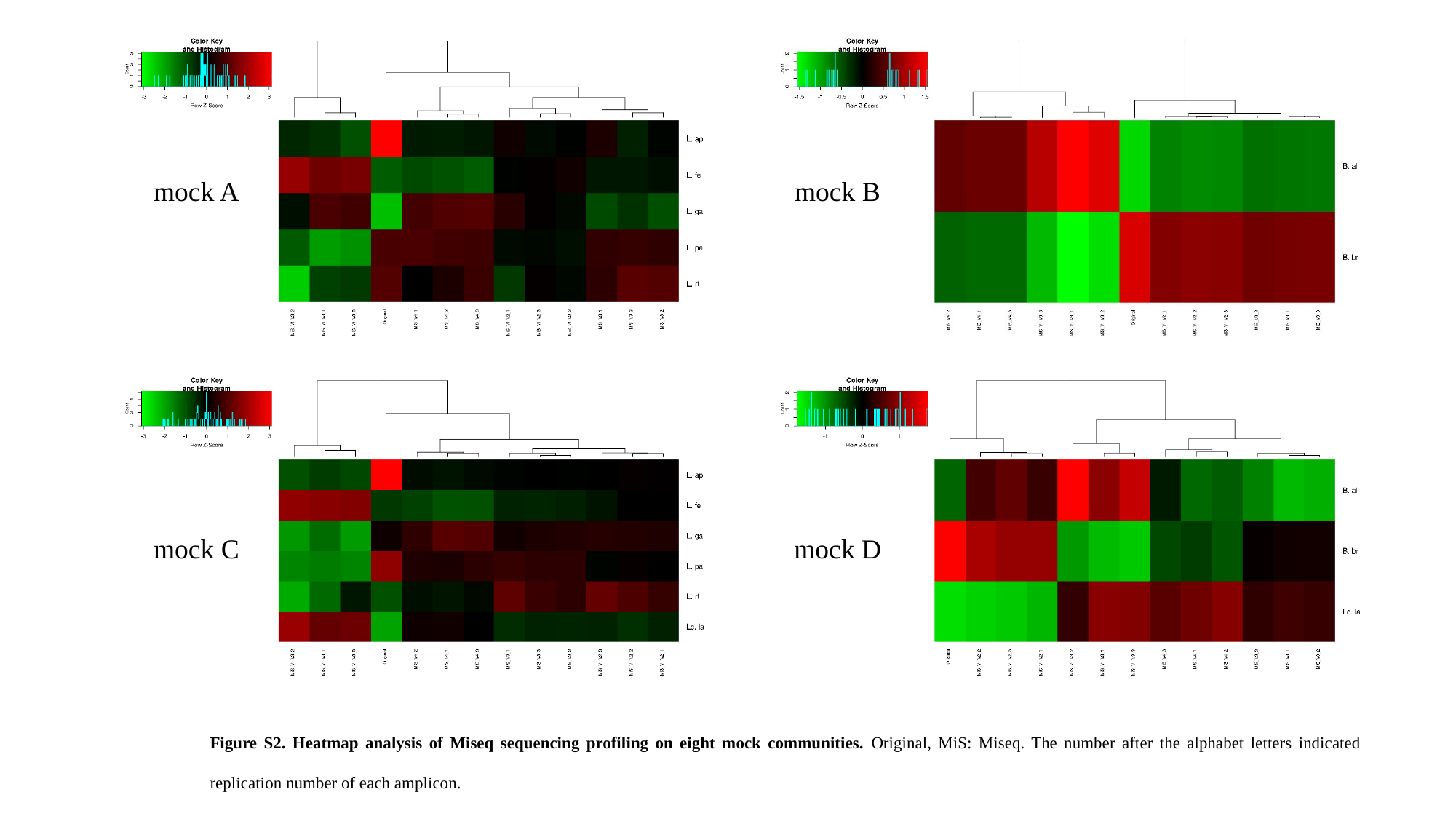

mock A
mock B
mock C
mock D
Figure S2. Heatmap analysis of Miseq sequencing profiling on eight mock communities. Original, MiS: Miseq. The number after the alphabet letters indicated replication number of each amplicon.

## Slide 4
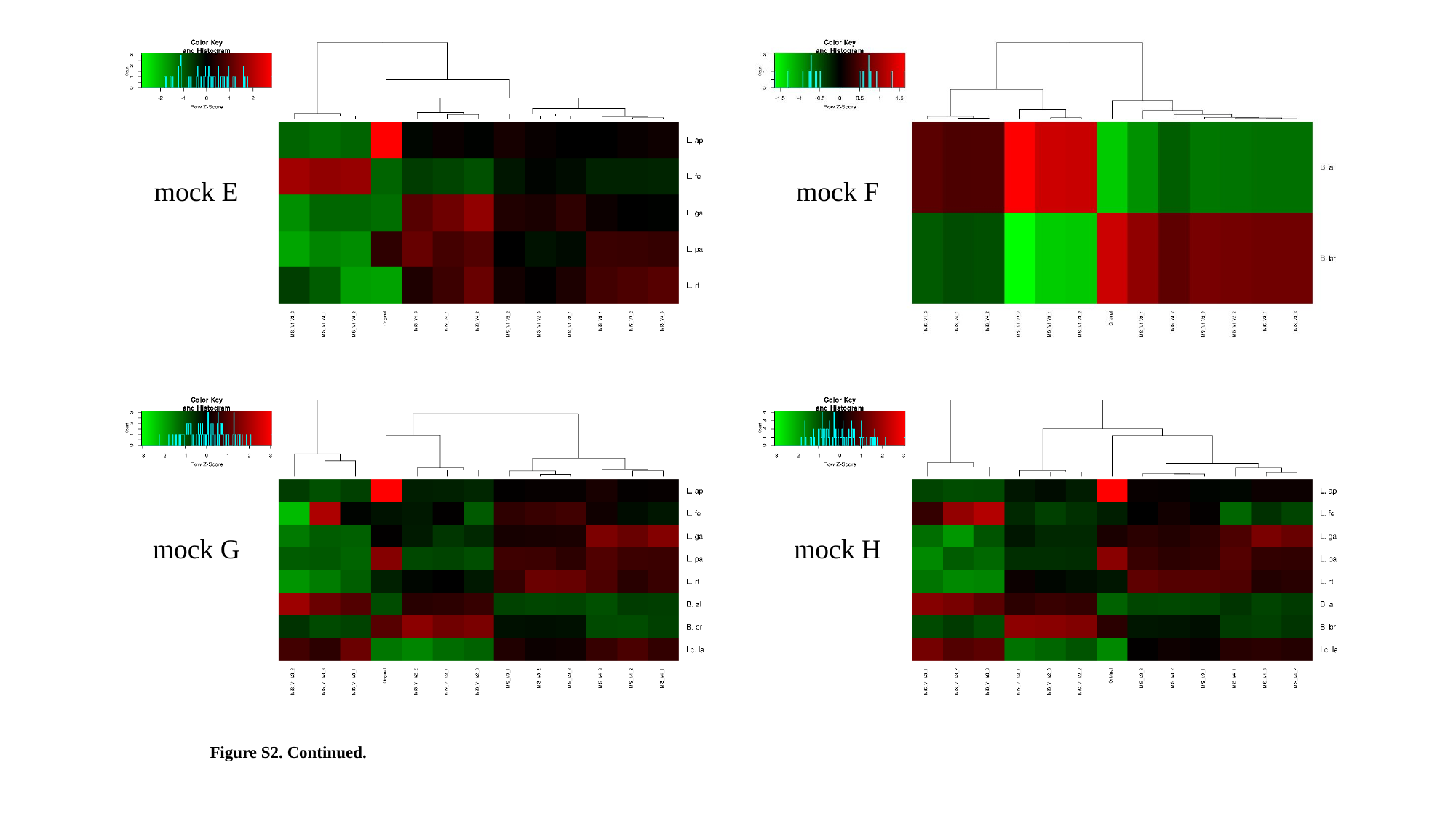

mock E
mock F
mock G
mock H
Figure S2. Continued.

## Slide 5
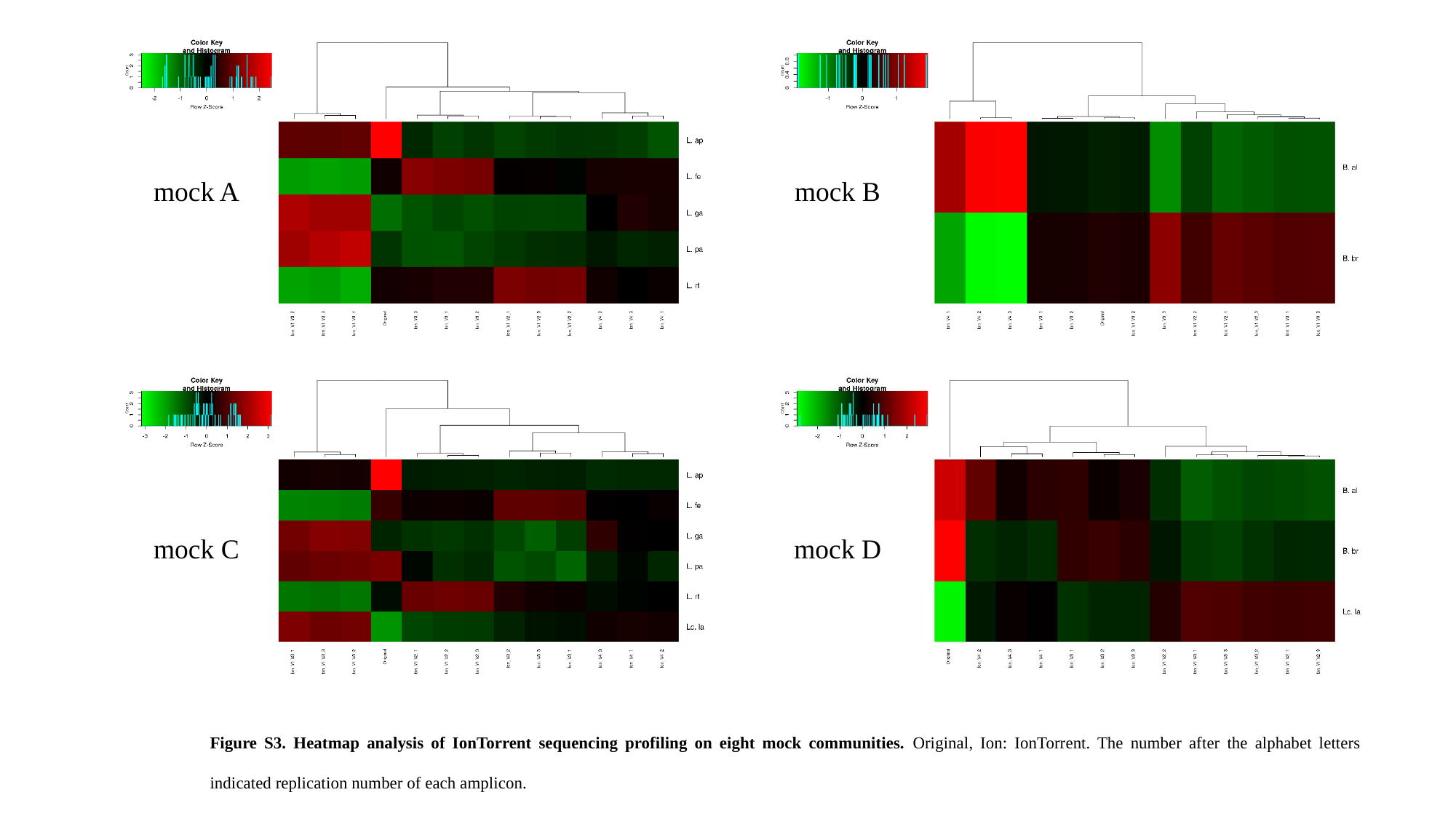

mock A
mock B
mock C
mock D
Figure S3. Heatmap analysis of IonTorrent sequencing profiling on eight mock communities. Original, Ion: IonTorrent. The number after the alphabet letters indicated replication number of each amplicon.

## Slide 6
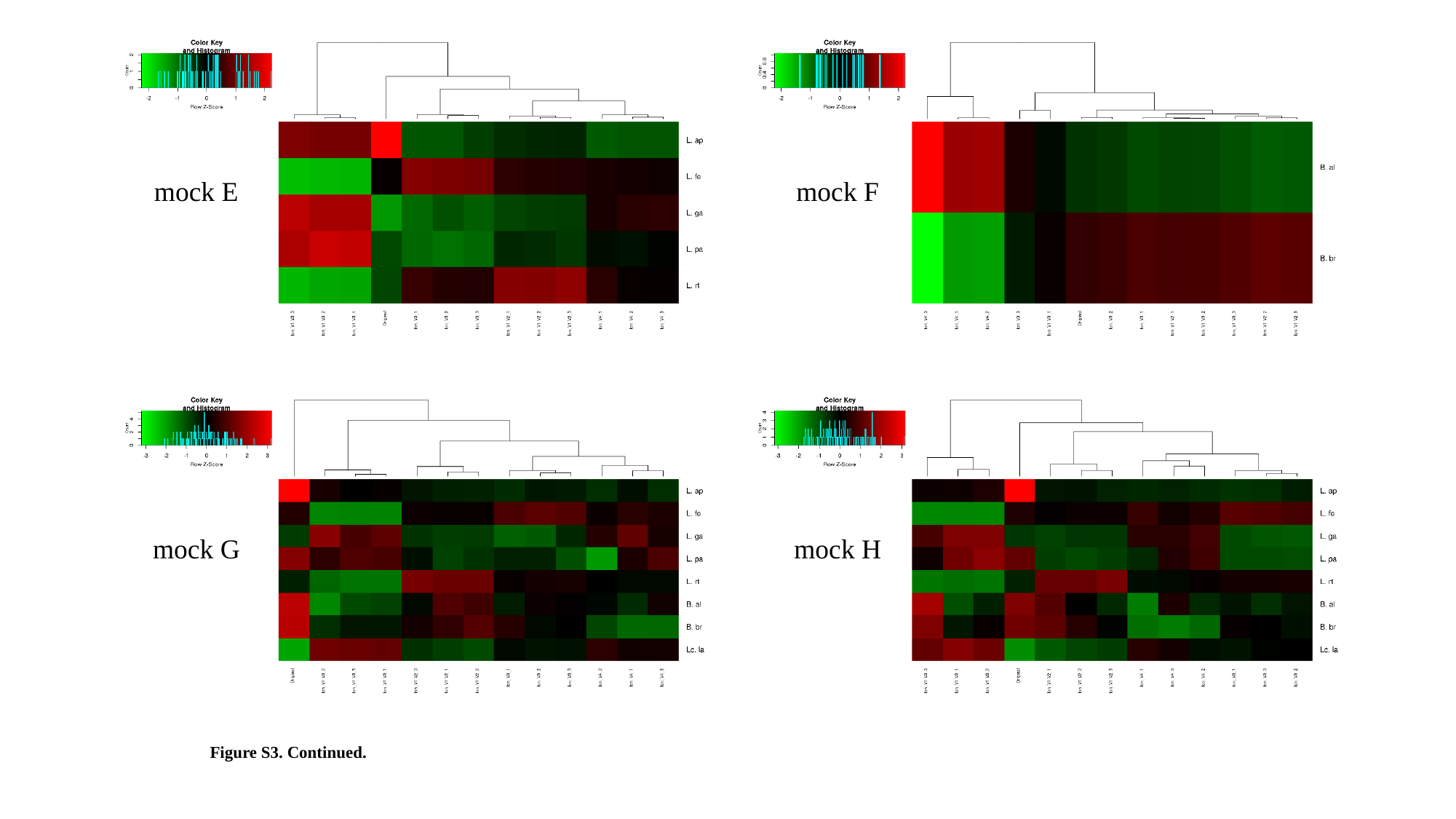

mock E
mock F
mock G
mock H
Figure S3. Continued.

## Slide 7
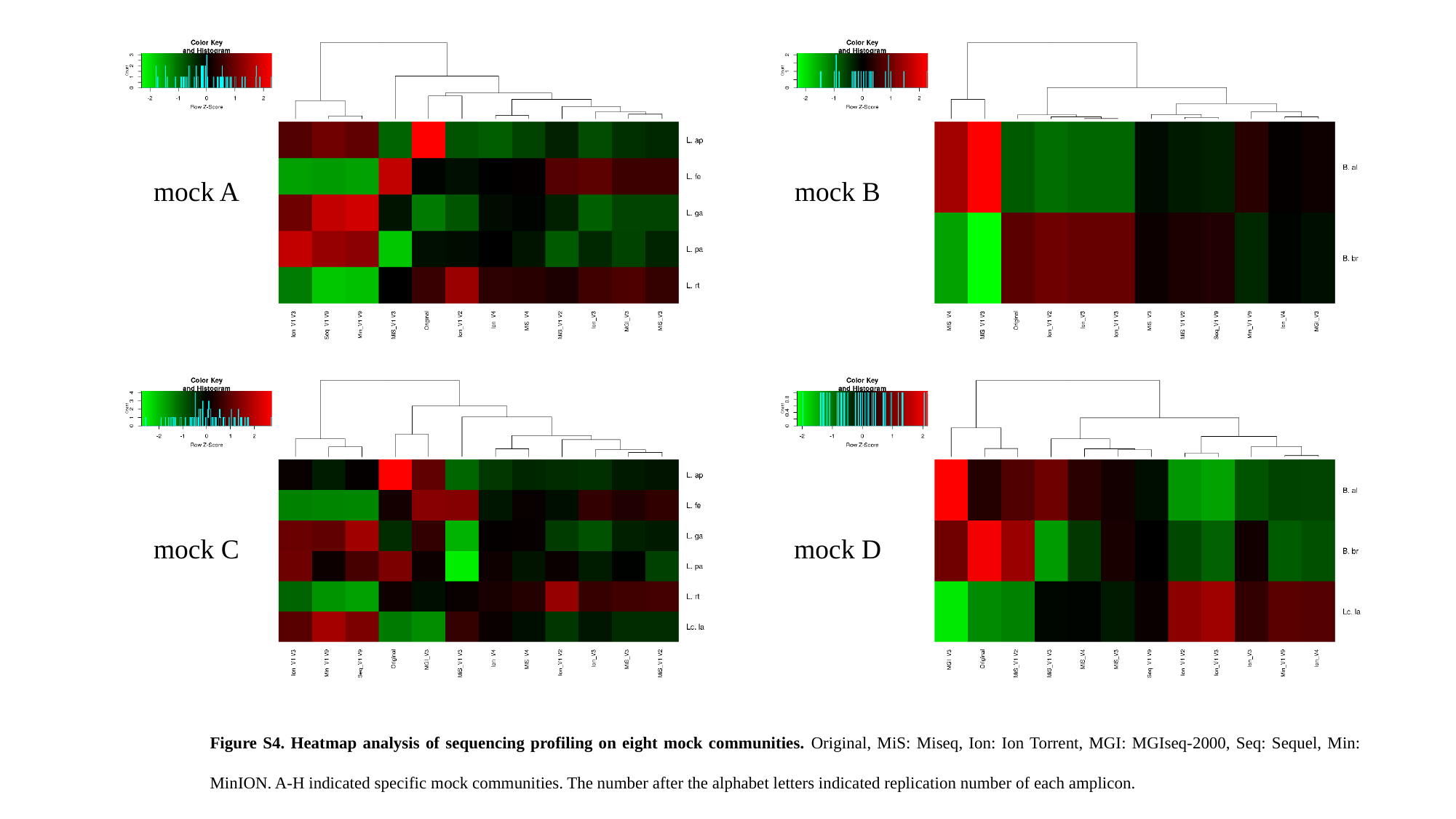

mock A
mock B
mock C
mock D
Figure S4. Heatmap analysis of sequencing profiling on eight mock communities. Original, MiS: Miseq, Ion: Ion Torrent, MGI: MGIseq-2000, Seq: Sequel, Min: MinION. A-H indicated specific mock communities. The number after the alphabet letters indicated replication number of each amplicon.

## Slide 8
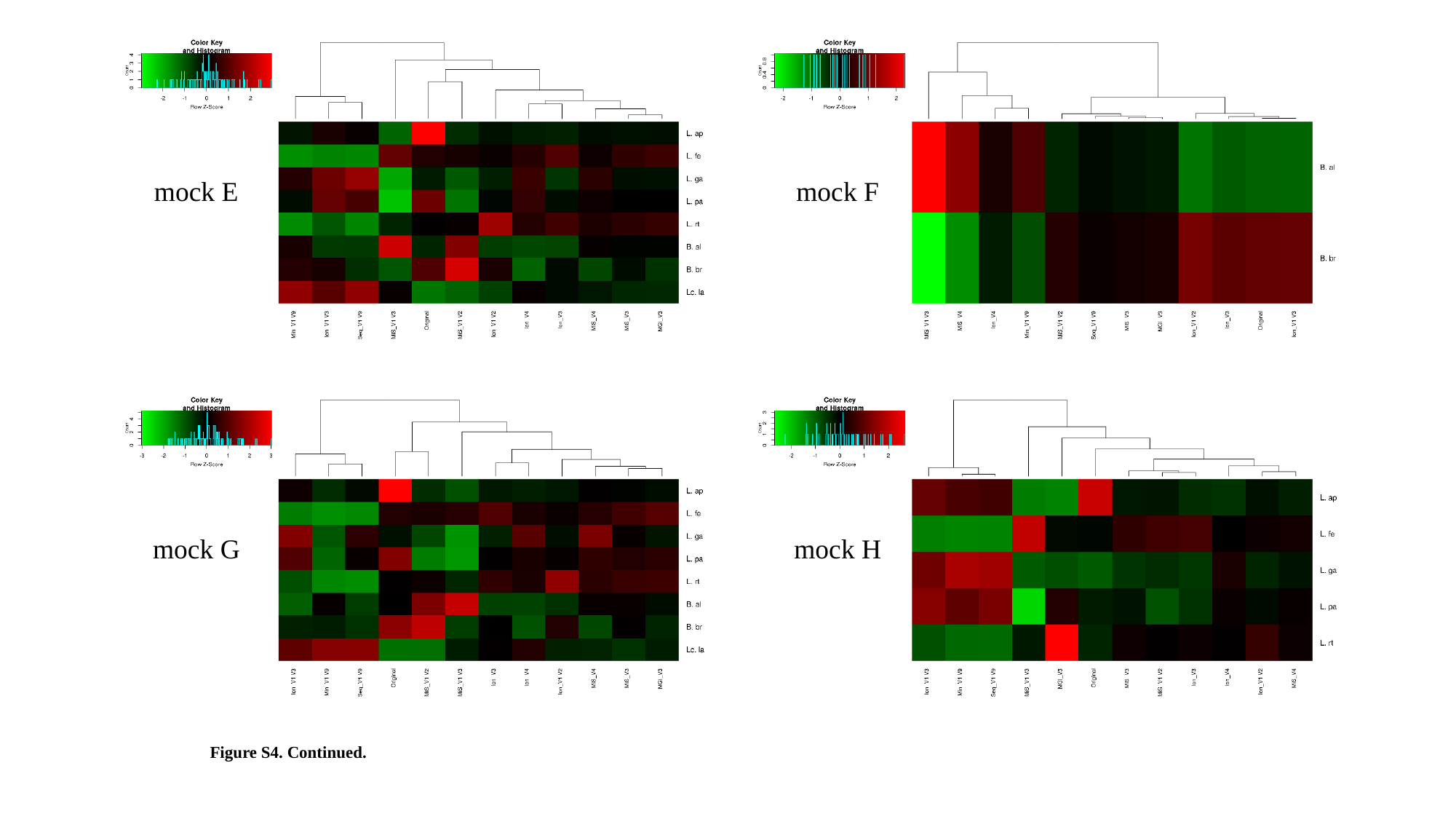

mock E
mock F
mock G
mock H
Figure S4. Continued.
